# Leptin Effects on the Regenerative Capacity of Human Periodontal Cells

**DOI:** 10.1155/2014/180304

**Published:** 2014-07-22

**Authors:** Marjan Nokhbehsaim, Sema Keser, Andressa Vilas Boas Nogueira, Andreas Jäger, Søren Jepsen, Joni Augusto Cirelli, Christoph Bourauel, Sigrun Eick, James Deschner

**Affiliations:** ^1^Experimental Dento-Maxillo-Facial Medicine, University of Bonn, 53111 Bonn, Germany; ^2^Clinical Research Unit 208, University of Bonn, 53111 Bonn, Germany; ^3^Department of Diagnosis and Surgery, School of Dentistry, UNESP, 14801-903 Araraquara, SP, Brazil; ^4^Department of Orthodontics, University of Bonn, 53111 Bonn, Germany; ^5^Department of Periodontology, Operative and Preventive Dentistry, University of Bonn, 53111 Bonn, Germany; ^6^Oral Technology, Center of Dento-Maxillo-Facial Medicine, University of Bonn, 53111 Bonn, Germany; ^7^Department of Periodontology, Laboratory of Oral Microbiology, University of Bern, 3010 Bern, Switzerland

## Abstract

Obesity is increasing throughout the globe and characterized by excess adipose tissue, which represents a complex endocrine organ. Adipose tissue secrets bioactive molecules called adipokines, which act at endocrine, paracrine, and autocrine levels. Obesity has recently been shown to be associated with periodontitis, a disease characterized by the irreversible destruction of the tooth-supporting tissues, that is, periodontium, and also with compromised periodontal healing. Although the underlying mechanisms for these associations are not clear yet, increased levels of proinflammatory adipokines, such as leptin, as found in obese individuals, might be a critical pathomechanistic link. The objective of this study was to examine the impact of leptin on the regenerative capacity of human periodontal ligament (PDL) cells and also to study the local leptin production by these cells. Leptin caused a significant downregulation of growth (TGF*β*1, and VEGFA) and transcription (RUNX2) factors as well as matrix molecules (collagen, and periostin) and inhibited SMAD signaling under regenerative conditions. Moreover, the local expression of leptin and its full-length receptor was significantly downregulated by inflammatory, microbial, and biomechanical signals. This study demonstrates that the hormone leptin negatively interferes with the regenerative capacity of PDL cells, suggesting leptin as a pathomechanistic link between obesity and compromised periodontal healing.

## 1. Introduction

The prevalence of overweight and obesity has been increasing throughout the globe over the past decades [1, 2]. According to a recent survey conducted in Germany from 2008 through 2011, 67.1% of men and 53.0% of women are overweight, and 23.3% of men and 23.9% of women are obese. Moreover, the prevalence of obesity has risen substantially in this population [3]. Since obesity is an important risk factor for many chronic diseases and conditions, such as type 2 diabetes mellitus, dyslipidemia, hypertension, cardiovascular diseases, and cancer, massive healthcare costs associated with the obesity epidemic have become a heavy financial burden for the economy and emerged as some of the most pressing global issues [1, 4–6].

Obesity is characterized by excess adipose tissue, in which adipocytes are increased in number and volume. Adipose tissue is considered a complex endocrine and highly active metabolic organ, which synthesizes and secretes a variety of bioactive molecules called adipokines [7–10]. These molecules act at both the systemic (endocrine) and the local (autocrine/paracrine) levels. Obesity causes an increase in the synthesis of proinflammatory adipokines, such as leptin, and a decrease in the production of anti-inflammatory adipokines, which results in the development of a chronic, low-grade inflammatory state [7–10].

Leptin is an adipose-derived hormone secreted in proportion to size and number of adipocytes. Therefore, plasma leptin levels are increased in obesity and decreased after weight loss [11]. Although leptin is mainly synthesized by adipose tissue, this adipokine is also produced at low levels in other tissues. Leptin inhibits appetite, stimulates energy expenditure, and modulates lipid and bone metabolism, coagulation, hematopoiesis, function of pancreatic beta-cells, and insulin sensitivity [8, 12]. Moreover, leptin regulates the immune system and inflammatory response, with mainly proinflammatory actions [8, 12–15].

Leptin mediates its effects by binding to the full-length leptin receptor (LEPR). In addition to brain, LEPR is also expressed in a wide range of peripheral tissues. Upon binding of leptin to its receptor, a number of intracellular pathways and transcription factors are activated [12–16].

Recent meta-analyses have revealed that obesity is associated with periodontitis, a chronic disease characterized by the irreversible destruction of the tooth-supporting tissues, that is, periodontium [17, 18]. The periodontium consists of the gingiva, periodontal ligament (PDL), root cementum, and alveolar bone. Pathogenic bacteria, such as* Fusobacterium nucleatum*,* Porphyromonas gingivalis*, and* Treponema denticola* in the subgingival dental plaque, are essential for the initiation and progression of periodontitis [19, 20]. However, cofactors, such as smoking, genetic predisposition, or occlusal loading, can also contribute to the development of periodontitis [21]. The pathogenic bacteria provoke an inflammatory host response, which involves inflammatory molecules, such as interleukin (IL)-1*β*, in the periodontal tissues. If the inflammatory process is exaggerated and sustained, matrix degradation and bone resorption, formation of periodontal pockets, and even tooth loss can occur [21, 22].

Periodontitis can be successfully treated. By reducing or eliminating the pathogenic microorganisms in the periodontal pockets, periodontal therapy seeks to arrest inflammation and tissue destruction [23]. Regeneration of lost periodontal structures can be stimulated by the application of bioactive molecules, such as enamel matrix derivative (EMD), during periodontal surgery [24–26]. EMD induces the production of several growth factors, such as transforming growth factor (TGF) *β*1 and vascular endothelial growth factor (VEGF) A, matrix molecules, such as collagen type I (COL1) and periostin (POSTN), and osteogenesis-related factors, such as runt-related transcription factor (RUNX) 2, and accelerates in vitro wound healing [27, 28]. The regeneration-promotive effects of EMD are mediated, at least in part, by bone morphogenetic protein (BMP) and TGF*β*, which trigger SMAD (sma- and mad-related protein) and non-SMAD signaling cascades [29–33]. A number of studies have shown that microbial, inflammatory, and biomechanical signals can interfere with the beneficial effects of EMD on periodontal cells, emphasizing the critical role of the cell environment for optimal periodontal regeneration [34–36].

Interestingly, obesity also negatively affects the response to periodontal therapy [37, 38]. The underlying mechanisms are not clear yet and increased serum levels of proinflammatory adipokines, as found in obese individuals, might be a critical pathomechanistic link in the association between obesity and compromised periodontal healing. Therefore, the objective of this in vitro study was to examine the impact of the proinflammatory hormone leptin on the regenerative capacity of PDL cells and to study the local production of leptin and its full-length receptor by these cells.

## 2. Materials and Methods

### 2.1. Culture and Treatment of Cells

Human PDL cells were derived from 21 periodontally healthy donors (mean age: 16.0 ± 1.4 years, min-max: 10–42 years; gender: 6 male/15 female; all normal weight), who had to undergo tooth extraction for orthodontic reasons. Written informed consent and approval of the Ethics Committee of the University of Bonn were obtained. Cells were derived from PDL explants as described elsewhere [39, 40]. Briefly, PDL tissue explants were dissected from the middle third of the root surface with a sharp scalpel then washed with phosphate-buffered saline (PBS, Sigma-Aldrich, Munich, Germany), minced into small pieces, and, finally, cultured in Dulbecco's modified essential medium (DMEM, Invitrogen, Karlsruhe, Germany) supplemented with 10% fetal bovine serum (FBS, Invitrogen), 100 units penicillin, and 100 *μ*g/mL streptomycin (Invitrogen) at 37°C in a humidified atmosphere of 5% CO_2_ for 2 to 4 weeks. Afterwards, cells were transferred into flasks for continued growth and phenotyped with the use of osteogenic markers [40]. Cells between 3rd and 5th passage were seeded (5,000 cells/cm^2^) on culture plates and grown to 80% confluence. One day prior to the experiments, the FBS concentration was reduced to 1%. Medium was changed every other day. In order to investigate the effects of leptin, various concentrations of this adipokine (1, 3, and 10 ng/mL; R&D Systems, Minneapolis, MN, USA) were added to cells. The leptin concentrations used in the present study are in the physiological range and have been measured in GCF from obese individuals [41]. To mimic regenerative conditions in vitro, cells were incubated with EMD (Emdogain, Straumann, Freiburg, Germany) at a concentration of 100 *μ*g/mL. In the present study, the commercially available product Emdogain, which consists of enamel matrix derivative and the vehicle propylene glycol alginate, was used. Our previous experiments revealed that the effects of Emdogain on the cell functions examined in the present study were identical to those of enamel matrix derivative without the vehicle (unpublished data). In order to simulate an inflammatory environment, cells were incubated with IL-1*β* (1 ng/mL; Calbiochem, San Diego, CA, USA). An infectious environment was mimicked by stimulating cells with the inactivated oral periodontopathogens* F. nucleatum* ATCC 25586,* P. gingivalis* ATCC 33277, or* T. denticola* ATCC 35405 (optical density: 0.1). Bacteria were suspended in PBS (OD_660_ nm = 1, equivalent to 1.2 × 10^9^ bacterial cells/mL) and subjected two times to ultrasonication (160 W for 15 min) resulting in a complete killing. Biomechanical loading conditions were accomplished by application of cyclic tensile strain (CTS, 20%) at a rate of 0.05 Hz to cells by using a strain device (CESTRA) developed at the University of Bonn. The strain regimen and the aforementioned concentrations had also been used in our previous experiments and ensured that data were comparable [42–47]. In order to unravel the intracellular mechanisms exploited by leptin to modulate the actions of EMD, cells were preincubated with a specific inhibitor against the NF*κ*B signaling pathway (pyrrolidine dithiocarbamate, PDTC; 10 *μ*M; Calbiochem) 1 h prior to experiments.

### 2.2. In Vitro Wound Healing

In order to study the effects of leptin on wound fill, an established in vitro wound healing model was used, as described in our previous experiments [44, 45, 47]. Briefly, cells were grown until confluence and 3 mm wide wounds, that is, cell-free areas, were created in a standardized manner in the cell monolayers. The wounded monolayers were treated with leptin (3 ng/mL) in the presence and absence of EMD for 4 d. Every day, the wounds were documented by inverse microscopy (Axiovert 25 C, 5x objective, Carl Zeiss, Oberkochen, Germany) and digital photography (Kodak DC 290, Kodak, Stuttgart, Germany). Afterwards, measurement and analysis of the wound widths were performed with special software (Alpha DigiDoc 1000, Alpha Innotech, San Leandro, CA, USA).

### 2.3. Real-Time PCR

RNA was extracted by using an RNA extraction kit (Qiagen, Hilden, Germany), and a total of 1 *μ*g of RNA was reverse transcribed using iScript Select cDNA Synthesis Kit (Bio-Rad Laboratories, Munich, Germany) at 42°C for 90 min followed by 85°C for 5 min. Expression of TGF*β*1, VEGFA, COL1, POSTN, RUNX2, Ki67, TGF*β* receptors (TGF*β*R1, TGF*β*R2), BMP receptors (BMPR1A, BMPR1B, and BMPR2), leptin, full-length LEPR, IL-6, tumor necrosis factor (TNF) *α*, cyclooxygenase (COX) 2, and glyceraldehyde-3-phosphate dehydrogenase (GAPDH) was detected by real-time PCR using the iCycler iQ detection system (Bio-Rad Laboratories), SYBR Green (Bio-Rad Laboratories), and specific primers (QuantiTect Primer Assay, Qiagen). One *μ*L of cDNA was amplified as a template in a 25 *μ*L reaction mixture containing 12.5 *μ*L 2x QuantiFast SYBR Green PCR Master Mix (Qiagen), 2.5 *μ*L of primers (0.5 *μ*M each), and 9 *μ*L deionized water. The mixture was heated initially at 95°C for 5 min and then followed by 40 cycles with denaturation at 95°C for 10 s and combined annealing/extension at 60°C for 30 s. GAPDH was used as an endogenous control. The data were analyzed by the comparative threshold cycle method.

### 2.4. ELISA

Protein levels of TGF*β*1, VEGFA, and leptin released from the cells into the medium and LEPR protein in cell lysates were measured by commercially available enzyme-linked immunosorbent assay (ELISA) kits (R&D Systems) according to the manufacturer's instructions. The absorbance was analyzed by using a microplate reader (PowerWave x, BioTek Instruments, Winooski, VT, USA) at 450 nm. Data were normalized by cell number determined with an automatic cell counter (Moelab, Hilden, Germany).

### 2.5. Immunofluorescence

Cells were fixed with 4% paraformaldehyde in PBS pH 7.4 for 10 min, washed with PBS (Sigma-Aldrich, Munich, Germany), and treated with 0.1% Triton X-100 (Sigma-Aldrich) for 5 min. Afterwards, cells were washed again and blocked with nonfat dry milk (Bio-Rad Laboratories) for 1 h. After washing, cells were incubated with primary rabbit anti-SMAD1/5/8 (1 : 200; Santa Cruz Biotechnology, Santa Cruz, CA, Germany) or anti-NF*κ*B p65 (1 : 400; Cell Signalling Technology, Danvers, MA, USA) antibodies for 90 min and with CY3-conjugated goat anti-rabbit IgG (1 : 2,000; Abcam, Cambridge, MA, USA) for 45 min. Cells were observed under a 20x objective using an Axioplan 2 imaging microscope (Carl Zeiss). The images were captured with a PVCAM camera and the VisiView capturing software (Visitron Systems, Puchheim, Germany).

### 2.6. Statistical Analysis

All experiments were performed in triplicate and repeated at least twice. Mean values and standard errors of the mean (SEM) were calculated. Parametric (ANOVA followed by Dunnett's or Tukey's tests) and nonparametric (Mann-Whitney *U*) tests were applied for statistical analysis by using the IBM SPSS Statistics 22 software (IBM Corporation, Armonk, NY, USA). Differences between groups were considered significant at *P* < 0.05.

## 3. Results

### 3.1. Inhibition of EMD Effects by Leptin

**Figure 1:**
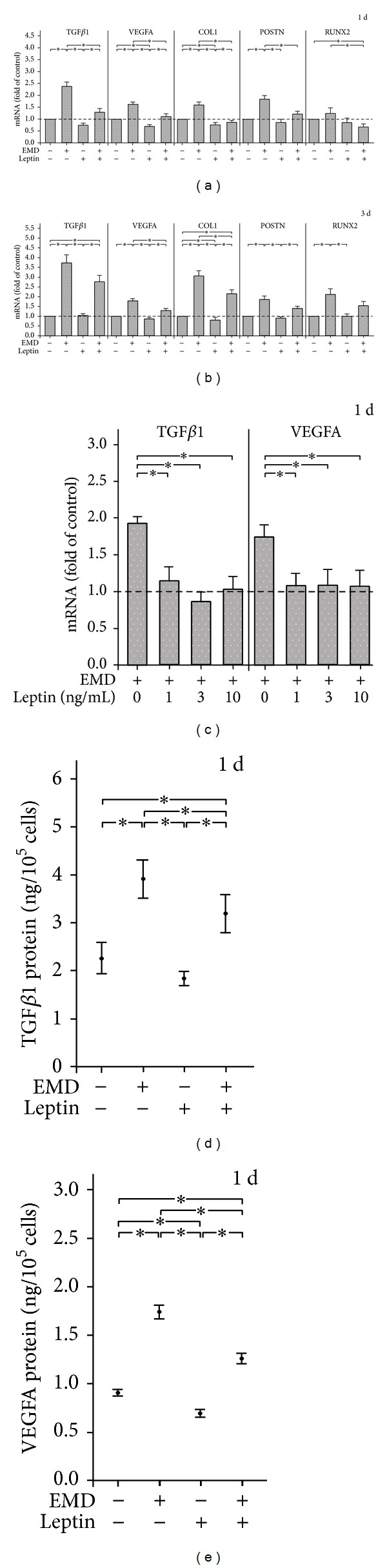
Effect of EMD and/or leptin (3 ng/mL) on TGF*β*1, VEGFA, COL1, POSTN, and RUNX2 mRNA at 1 d (a) and 3 d (b). Untreated cells served as control. Mean ± SEM (*n* = 18); *significant (*P* < 0.05) difference between groups. Effect of various concentrations (0, 1, 3, and 10 ng/mL) of leptin on TGF*β*1 and VEGFA mRNA in EMD-treated cells at 1 d (c). Mean ± SEM (*n* = 9); *significant (*P* < 0.05) difference between groups. Effect of EMD and/or leptin (3 ng/mL) on protein levels of TGF*β*1 (d) and VEGFA (e) released from cells into the medium at 1 d. Untreated cells served as control. Mean ± SEM (*n* = 18); *significant (*P* < 0.05) difference between groups.

EMD caused a significant increase in the TGF*β*1, VEGFA, COL1, and POSTN expressions and tended to upregulate RUNX2 at 1 d, as shown in Figure 1(a). Interestingly, when EMD-treated cells were concomitantly exposed to leptin, the EMD-induced TGF*β*1, VEGFA, COL1, POSTN, and RUNX2 expressions were significantly reduced at this time point (Figure 1(a)). A significant upregulation of TGF*β*1, VEGFA, COL1, POSTN, and RUNX2 expressions was also found at 3 d (Figure 1(b)). At this time point, leptin inhibited significantly the EMD-induced VEGFA and COL1 expressions and also showed a tendency to decrease the EMD-induced TGF*β*1, POSTN, and RUNX2 expressions (Figure 1(b)). As depicted in Figures 1(c)–1(e), the inhibitory effects of leptin on EMD actions were observed for different concentrations of this adipokine and also at protein level, as evidenced by reduced TGF*β*1 and VEGFA protein levels in the presence of leptin. In the absence of EMD, leptin only inhibited significantly the VEGFA production at 1 d (Figures 1(a) and 1(e)) and the COL1 expression at 1 and 3 d (Figures 1(a) and 1(b)).

**Figure 2:**
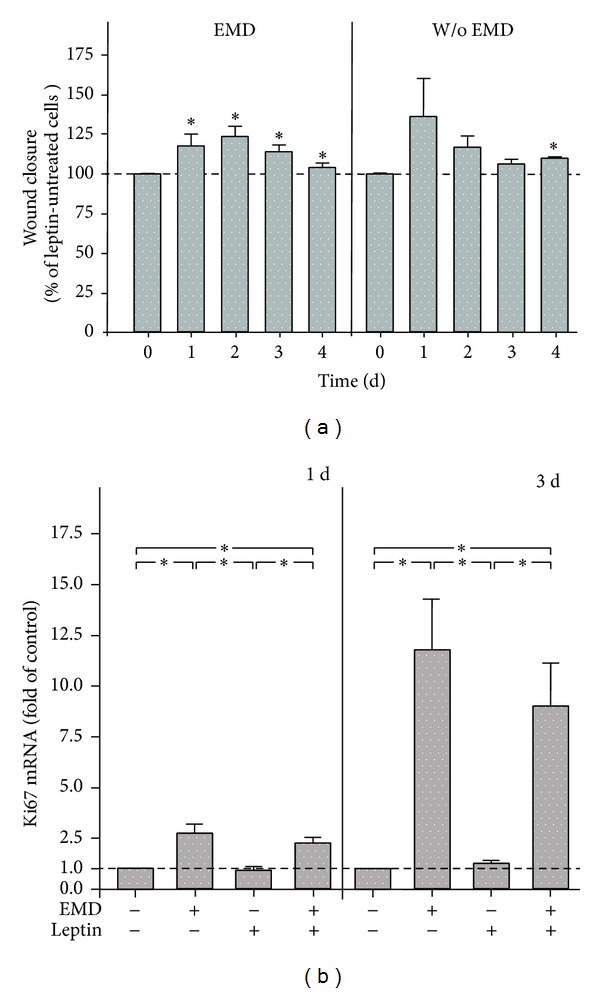
Effect of leptin (3 ng/mL) on wound closure in the presence and absence of EMD (a). The wound closure, that is, the percentage of fill of the initially cell free zones created by wounding, was analyzed over 4 d. Mean ± SEM (*n* = 12); *significantly (*P* < 0.05) different from leptin-untreated cells. Effect of EMD and/or leptin (3 ng/mL) on Ki67 mRNA at 1 d and 3 d (b). Untreated cells served as control. Mean ± SEM (*n* = 18); *significant (*P* < 0.05) difference between groups.

In an in vitro wound healing model, the impact of leptin on wound closure in the presence and absence of EMD was studied. In the presence of EMD, leptin increased significantly the wound closure at 1, 2, 3, and 4 d, as compared to leptin-untreated cell cultures, as shown in Figure 2(a). In the absence of EMD, leptin tended to stimulate the wound closure at 1, 2, and 3 d and increased significantly the wound closure at 4 d (Figure 2(a)). Since the wound closure results from proliferation and migration in this model, we further studied the influence of leptin on proliferation. As expected, EMD caused an increase in Ki67, a marker of proliferation, at 1 and 3 d (Figure 2(b)). However, the EMD-upregulated Ki67 expression was not significantly affected by leptin at both time points. Leptin had also no impact on the cell numbers in the presence or absence of EMD (data not shown).

### 3.2. Effect of Leptin on Intracellular Signaling

**Figure 3:**
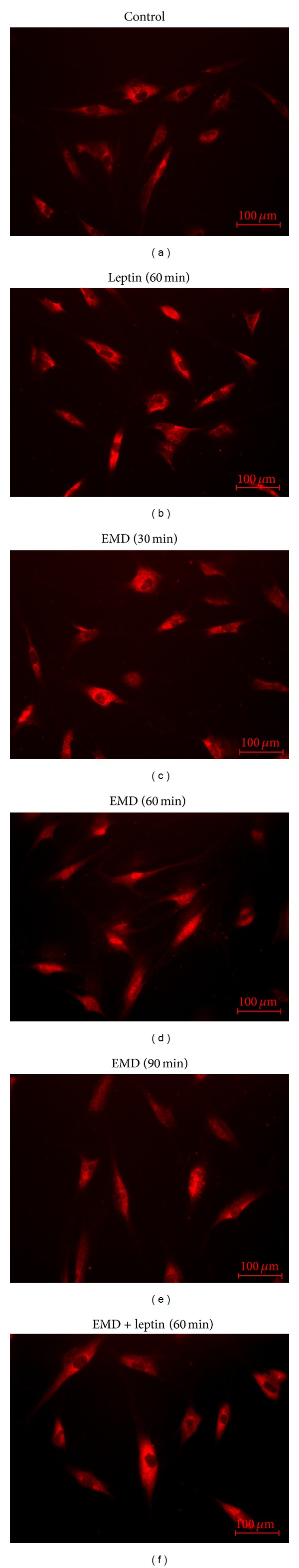
Effect of EMD on the nuclear translocation of SMAD1/5/8 in the presence and absence of leptin (3 ng/mL), as determined by immunofluorescence ((a)–(f)). Experiments were performed in triplicate and repeated twice. Untreated cells served as control. Images from one representative donor are shown.

EMD induced a nuclear translocation of SMAD1/5/8, which was most pronounced at 60 min. However, in the presence of leptin, the EMD-stimulated SMAD1/5/8 nuclear translocation was almost completely blocked, as shown in Figures 3(a)–3(f).

**Figure 4:**
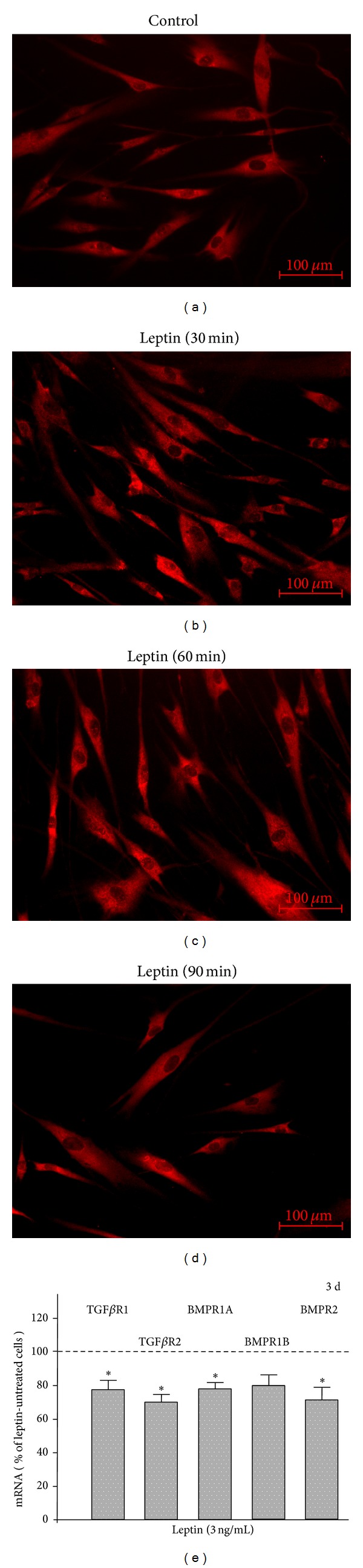
Effect of leptin (3 ng/mL) on the nuclear translocation of NF*κ*B p65, as examined by immunofluorescence ((a)–(d)). Experiments were performed in triplicate and repeated twice. Untreated cells served as control. Images from one representative donor are shown. Effect of leptin (3 ng/mL) on TGF*β*R1, TGF*β*R2, BMPR1A, BMPR1B, and BMPR2 mRNA at 3 d (e). Untreated cells served as control. Mean ± SEM (*n* = 18); *significantly (*P* < 0.05) different from control.

Next, we sought to examine a possible involvement of NF*κ*B in the inhibitory actions of leptin on EMD. When cells were incubated with a specific inhibitor of NF*κ*B signaling, the leptin-induced inhibition of the EMD-stimulated expression of TGF*β*1, VEGFA, and COL1 was abrogated by 95%, 29%, and 100%, respectively, at 1 d, as analyzed by real-time PCR. Interestingly, leptin did not cause a remarkable NF*κ*B nuclear translocation over 90 min, as evidenced by immunofluorescence (Figures 4(a)–4(d)).

### 3.3. Impact of Leptin on Receptors for TGF*β* and BMP

Since EMD contains TGF*β*- and BMP-like activity and, therefore, exploits receptors for these growth and differentiation factors, we next studied the effects of leptin on TGF*β*R1, TGF*β*R2, BMPR1A, BMPR1B, and BMPR2. Although there were no significant effects on these receptors at 1 d, leptin reduced significantly the TGF*β*R1, TGF*β*R2, BMPR1A, and BMPR2 expressions at 3 d (Figure 4(e)).

### 3.4. Regulation of Leptin and Its Receptor in PDL Cells

**Figure 5:**
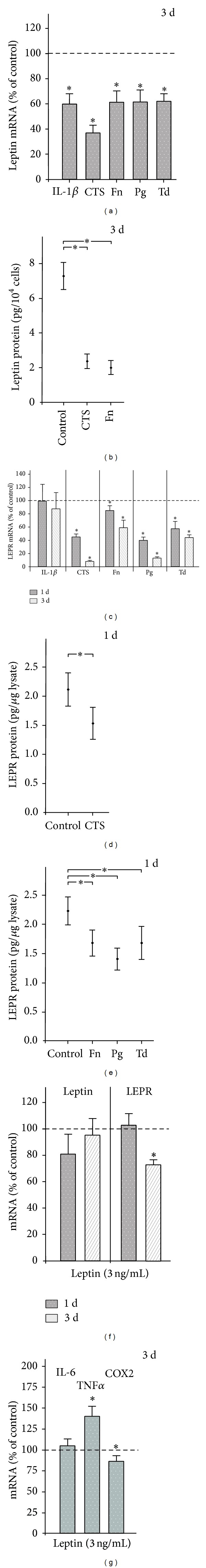
Effect of interleukin (IL)-1*β*, cyclic tensile strain (CTS),* F. nucleatum* (*Fn*),* P. gingivalis* (*Pg*), and* T. denticola* (*Td*) on leptin mRNA in PDL cells at 3 d (a). Untreated cells served as control. Mean ± SEM (*n* = 9); *significantly (*P* < 0.05) different from control. Effects of CTS and* Fn* on the protein level of leptin released from cells into the medium at 3 d (b). Untreated cells served as control. Mean ± SEM (*n* = 18); *significant (*P* < 0.05) difference between groups. Effects of IL-1*β*, CTS,* Fn*,* Pg*, and* Td* on leptin receptor (LEPR) mRNA in PDL cells at 1 d and 3 d (c). Untreated cells served as control. Mean ± SEM (*n* = 9); *significantly (*P* < 0.05) different from control. Effects of CTS (d) and* Fn*,* Pg*, and* Td* (e) on LEPR protein in lysates from PDL cells at 1 d. Untreated cells served as control. Mean ± SEM (*n* = 18); *significant (*P* < 0.05) difference between groups. Effect of leptin (3 ng/mL) on its own mRNA and that of its receptor in PDL cells at 1 d and 3 d (f). Untreated cells served as control. Mean ± SEM (*n* = 18); *significantly (*P* < 0.05) different from control. Effect of leptin (3 ng/mL) on IL-6, tumor necrosis factor (TNF) *α*, and cyclooxygenase (COX) 2 mRNA in PDL cells at 3 d (g). Untreated cells served as control. Mean ± SEM (*n* = 18); *significantly (*P* < 0.05) different from control.

Next, we sought to examine if and under what conditions leptin and LEPR are produced in PDL cells. Leptin was constitutively expressed and this constitutive expression was significantly downregulated by IL-1*β*, CTS,* F. nucleatum*,* P. gingivalis*, and* T. denticola* at 1 d (data not shown) and 3 d (Figure 5(a)). The inhibitory effects were also found at protein level, as evidenced by the reduced protein levels of leptin released from cells exposed to CTS or* F. nucleatum* at 3 d (Figure 5(b)). Similar results were also observed for LEPR. Although IL-1*β* had no significant effect on the receptor expression, CTS,* F. nucleatum*,* P. gingivalis*, and* T. denticola* inhibited significantly LEPR at both time points (Figure 5(c)). In addition, CTS,* F. nucleatum*,* P. gingivalis*, and* T. denticola* reduced significantly the cellular LEPR protein level as measured in cell lysates at 1 d (Figures 5(d) and 5(e)). Since our experiments revealed that leptin is also produced by PDL cells, we wondered how leptin would affect its own expression and that of its receptor. As shown in Figure 5(f), leptin had no effect on its own expression, but caused a significant reduction of LEPR expression at 3 d.

### 3.5. Effects of Leptin on Inflammatory Mediators

In order to clarify whether leptin exerts proinflammatory effects by upregulation of inflammatory mediators, we also studied the expression of IL-6, TNF*α*, and COX2 in PDL cells. Whereas leptin had no impact on all these molecules at 1 d and on IL-6 at 3 d, leptin caused a slight but significant TNF*α* upregulation and COX2 downregulation at 3 d (Figure 5(g)).

## 4. Discussion

**Figure 6:**
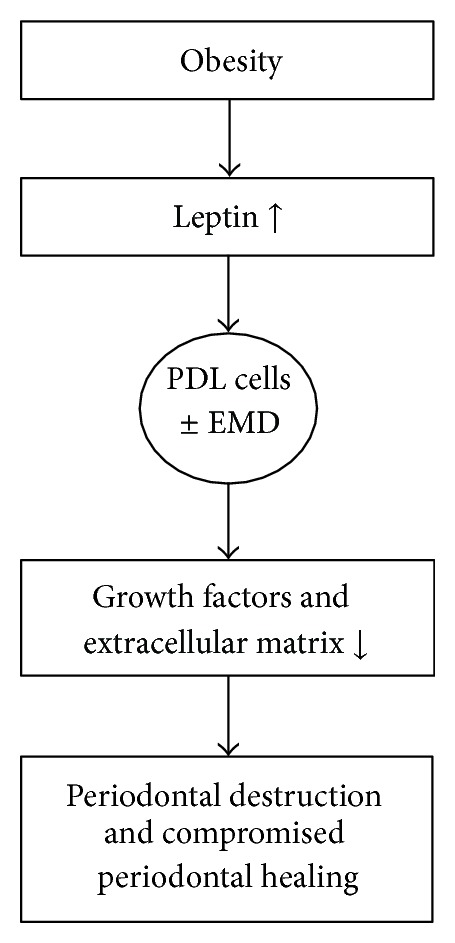
Hypothetical scheme illustrating the possible involvement of leptin in the relationship of obesity with periodontal destruction and compromised periodontal healing. The hormone leptin, whose plasma and gingival crevicular fluid levels are increased in obese individuals, inhibits the production of growth factors and extracellular matrix molecules by PDL cells under normal (absence of EMD) and regenerative (presence of EMD) conditions, which in turn could favor periodontal destruction and compromised periodontal healing, respectively.

The present study provides original evidence that the hormone leptin, whose plasma and gingival crevicular fluid (GCF) levels are increased in obese individuals, negatively interferes with the regenerative capacity of PDL cells (Figure 6) [11, 41]. A number of PDL cell functions critical for periodontal regeneration were significantly compromised in the presence of this adipokine. Our observation is in line with a previous study, in which visfatin, another proinflammatory adipokine, has also been shown to abrogate the regeneration-promotive effects of EMD on periodontal cells [47]. These studies underline the detrimental role of proinflammatory adipokines in periodontal healing and strongly suggest such adipokines as a pathomechanistic link between obesity and compromised periodontal healing. Future studies should also focus on the effects of leptin on other periodontal cells, such as gingival cells as well as osteo- and cementoblasts, since they are also critical to periodontal healing.

Our experiments revealed that leptin counteracted the stimulatory effects of EMD on the synthesis of growth and transcription factors as well as extracellular matrix molecules, which are strongly involved in periodontal regeneration. VEGFA promotes wound healing by its stimulatory effects on vascular permeability and recruitment of circulating neutrophils and monocytes to the site of injury. This growth factor enhances survival, proliferation, migration, and invasion of endothelial cells, thereby contributing to angiogenesis. In addition, VEGFA stimulates pericytes to coat and stabilize the vasculature [48–50]. Although leptin upregulates VEGFA in a number of cells, the VEGFA expression and release from PDL cells were reduced by leptin in our experiments [51, 52]. Interestingly, in human gingival biopsies, the leptin concentration decreases as the probing pocket depth increases. Moreover, the decrease in leptin is associated with an increase in VEGFA [53]. This negative correlation between leptin and VEGFA in human gingiva supports our finding and may suggest that leptin has no proangiogenic effects on periodontal tissues. However, further experiments are needed to clarify this point. TGF*β*, another important growth factor, comprises three isoforms and also promotes wound healing by its stimulatory effects on the migration, chemotaxis, and proliferation of monocytes/macrophages, fibroblasts, and endothelial cells, keratinocyte migration and reepithelialization, the synthesis of extracellular matrix molecules, and differentiation of stem cells [54–56]. For periodontal homeostasis and regeneration, matrix molecules, such as COL1 and POSTN, are also essential. COL1 is responsible for the mechanical characteristics of the periodontal connective tissues and provides tensile strength. POSTN promotes formation of high stiffness collagen through effective collagen cross-linking and also interacts with other extracellular matrix molecules. Moreover, POSTN aids in dispersing mechanical forces applied to the PDL and also plays a role in osteoblast adhesion, differentiation, and survival [57–59]. PDL cells can also undergo osteogenic differentiation, and several studies have demonstrated that EMD stimulates the expression of osteogenesis-associated factors, such as RUNX2, in PDL cells [27]. RUNX2 is a critical transcription factor in osteoblast commitment and differentiation and, thereby, bone formation [60]. Leptin abrogated the stimulatory actions of EMD on the aforementioned growth and transcription factors as well as matrix molecules, suggesting that leptin may interfere with the EMD-induced effects on both periodontal soft and hard tissue regeneration.

Interestingly, leptin did not compromise the in vitro wound healing, which is in clear contrast to our previous findings in PDL cells, which were exposed to visfatin [47]. Leptin enhanced the wound closure, especially in the presence of EMD. Since the in vitro wound healing may be affected by proliferation, we also studied the effect of leptin on Ki67, a marker of cell proliferation. As expected, EMD caused a significant Ki67 upregulation. However, leptin did not further enhance the EMD-induced Ki67 expression, suggesting that the beneficial effects of leptin on wound healing are not accomplished by stimulating PDL cell proliferation. Further studies should clarify if and how leptin impacts migration, which also determines the wound fill rate in this healing assay.

To the best of our knowledge, the present study shows for the first time that leptin inhibits SMAD signaling in EMD-treated PDL cells. As already shown in our previous studies, EMD, which has TGF*β*- and BMP-like activity, triggers the SMAD signaling pathway [44, 47]. Interestingly, leptin abrogated the stimulatory effect of EMD on the nuclear translocation of SMADs, similarly to visfatin. Therefore, the SMAD signaling pathway may be a critical target for the inhibitory actions of proinflammatory adipokines in PDL cells. When cells were preincubated with a specific inhibitor of NF*κ*B signaling, the leptin-induced inhibition of the EMD-stimulated upregulation of TGF*β*1, VEGFA, and COL1 was abrogated by 95%, 29%, and 100%, respectively, after 1 d. These results indicate that the NF*κ*B pathway is also involved in the counteracting effects of leptin on EMD. However, as evidenced by immunofluorescence, leptin did not cause a remarkable NF*κ*B nuclear translocation within 90 min, suggesting that NF*κ*B transactivation maybe be a later event and not caused directly by leptin. Further studies are needed to clarify how NF*κ*B interferes with SMAD signaling and whether additional intracellular signaling pathways are exploited by leptin for its inhibitory actions.

Our previous studies have shown that inflammatory and biomechanical signals can impact BMP and TGF*β* receptors [44, 45, 47, 61]. Since EMD contains BMP- and TGF*β*-like activity, we also studied the influence of leptin on these receptors. Interestingly, leptin caused a downregulation of the BMP and TGF*β* receptors by 20–30%, indicating that leptin may regulate actions of EMD also at receptor level.

Since obesity is characterized by leptin resistance, further studies should examine if the effects of leptin, as found in the present study, can be confirmed in PDL cells from obese individuals. Currently, we are studying the effects of obesity on periodontal regeneration in an established fenestration-type defect model in rats fed with normal or high-fat diet. This in-vivo study will help verify our in-vitro data and provide further insight into the role of obesity in periodontal homeostasis and healing.

A number of studies have shown that periodontitis results in elevated serum levels of leptin and that periodontal therapy can reduce serum leptin levels in periodontally-diseased patients [41, 62–65]. By contrast, leptin levels are decreased in GCF and gingival tissues in periodontitis patients, as compared to periodontally healthy individuals [53, 62, 66, 67]. In addition, smoking and biomechanical forces also lead to reduced leptin levels in GCF [68, 69]. These studies suggest that periodontitis, smoking, and biomechanical forces impact the local leptin synthesis in periodontium. However, only few studies have focused on the local production of leptin and its full-length receptor in periodontal tissues. These studies have demonstrated that leptin and its receptor are synthesized in PDL, gingival, and alveolar bone cells [70, 71]. Our study confirms that both leptin and its receptor are constitutively expressed in PDL cells. The leptin release from PDL cells might be lower than that from adipocytes [72, 73]. However, a comparison is difficult, because even the leptin secretion from adipocytes depends on a number of parameters, such as gender, subcutaneous or omental origin, and genotype [74, 75].

Our findings provide original evidence that inflammatory, microbial, and biomechanical signals cause a significant downregulation of leptin and its receptor. This observation is in accordance with the aforementioned clinical studies, which found decreased leptin levels in GCF from periodontally diseased or biomechanically loaded sites [62, 66, 67]. Although only PDL cells were used in our experiments, it can be speculated that the increased serum leptin levels observed in periodontitis do not result from an enhanced local production of leptin in the periodontium. Interestingly, our experiments did not reveal any autostimulation of leptin or its receptor. Further studies should clarify the regulation of leptin and its receptor in other periodontal cells and their contribution to the altered leptin levels in periodontitis.

It has been reported that leptin enhances the TNF*α* production in LPS-stimulated macrophages, confirming the proinflammatory characteristics of leptin [76]. Although leptin also increased the TNF*α* expression in our experiments, IL-6 was not regulated by leptin. Moreover, the expression level of COX2 was slightly, but significantly, downregulated by this adipokine. Further studies are needed to prove the proinflammatory nature of leptin in the PDL.

As in our previous experiments, IL-1*β* was used to simulate inflammatory conditions in vitro, because this proinflammatory cytokine is increased in GCF and gingival tissues at inflamed sites [42, 44, 46, 77–79]. In order to mimic microbial conditions in vitro, PDL cells were treated with a suspension of* F. nucleatum*,* P. gingivalis*, and* T. denticola*.* P. gingivalis* and* T. denticola* are gram-negative anaerobic bacteria and strongly linked to periodontitis [80–82].* F. nucleatum*, which is also a gram-negative microorganism, is associated with both gingivitis and periodontitis and acts as a bridge bacterium between early and late colonizers during plaque development [83–85]. Since periodontitis is caused by a complex bacterial biofilm, further studies should clarify whether leptin and its receptor are also regulated by other microorganisms, which are associated with periodontitis. The suspensions were subjected to intensive ultrasonication and contained disrupted cell wall particles with a high amount of LPS. However, additional microbial components may have been present in the suspensions. As in our previous studies, cells were subjected to tensile forces [43, 45, 61, 86]. However, during mastication, dental habits, and orthodontic treatment, the periodontium is subject to complex forces. Whether compressive, hydrostatic, and shear forces as well as their combinations exert similar effects on the production of leptin and its receptor has yet to be determined.

Taken together, our study shows for the first time that inflammatory, microbial, and biomechanical signals can inhibit the expression of leptin and its receptor in PDL cells, which may explain, at least in part, the reduced GCF leptin levels found in periodontitis and orthodontic patients. Furthermore, our experiments provide original evidence that the hormone leptin, whose plasma and GCF levels are increased in obese individuals, negatively interferes with the regenerative capacity of PDL cells, suggesting leptin as a pathomechanistic link between obesity and compromised periodontal healing.
